# A First-Principles Investigation of the Encapsulation of Nucleic-Acid-Derived Aromatic Heterocycles in Boron Nitride Nanotubes

**DOI:** 10.1021/acsomega.6c02732

**Published:** 2026-08-17

**Authors:** Thiago F. de Oliveira, Puspitapallab Chaudhuri, Cicero Mota, Hidembergue Ordozgoith da Frota, Angsula Ghosh

**Affiliations:** † Department of Materials Physics, 67892 Federal University of Amazonas, Manaus, Amazonas 69077-000, Brazil; ‡ Department of Mathematics, 67892 Federal University of Amazonas, Manaus, Amazonas 69077-000, Brazil

## Abstract

This work investigates the encapsulation of five nucleic-acid-derived aromatic heterocycles (nucleobases)- adenine, cytosine, guanine, thymine, and uracil- inside boron nitride nanotubes (BNNTs) to understand the fundamental mechanisms governing host–guest interactions in confined nanoscale environments. Four BNNTs with different chiralities and diameters, namely the zigzag (11,0) and (12,0) nanotubes and the armchair (6,6) and (7,7) nanotubes, were studied using periodic density functional theory calculations. The results indicate that encapsulation is energetically favorable for all heterocycle–BNNT combinations considered. The confinement process induces measurable structural distortions in the smaller-diameter nanotubes and modifies the electronic properties of the host nanotubes through local charge redistribution arising from the proximity of electronegative atoms in the encapsulated molecules. Among the systems investigated, adenine exhibits the most favorable formation energy. Analysis of the electronic structure reveals a systematic reduction of the BNNT band gap upon encapsulation, with uracil producing the largest effect and guanine the smallest relative to the pristine nanotubes. Effectively, the process of encapsulation transforms the insulating BNNTs into wide-gap semiconductors while preserving the confined molecules. These findings demonstrate that molecular confinement inside BNNT cavities can significantly influence the structural stability and electronic properties of the host nanotubes. The study provides fundamental insight into confinement-driven interactions between aromatic heterocycles and boron nitride nanotubes and contributes to a deeper understanding of host–guest behavior in nanoscale tubular materials.

## Graphical abstract

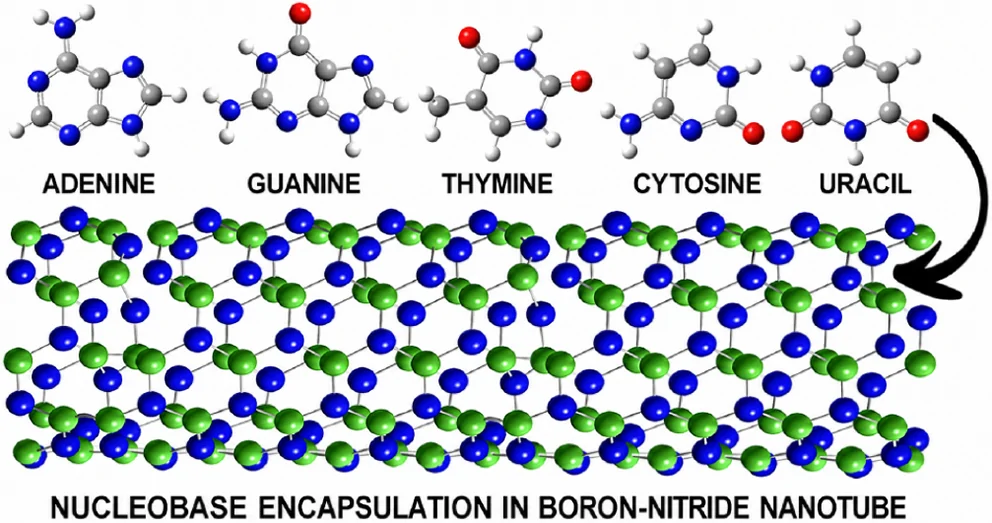

## 1 Introduction

The five nucleic-acid-derived aromatic heterocycles – adenine, guanine, cytosine, thymine, and uracil (hereafter referred to as nucleobases) – are the key components of the genetic macromolecules – deoxyribonucleic acid (DNA) and ribonucleic acid (RNA). These nitrogenous bases are essential to all biological processes, including cellular replication, protein synthesis, and heredity. Because of their well-defined aromatic structure and rich hydrogen-bonding capabilities, nucleobases have also attracted significant interest in recent years. In particular, their interaction with different forms of nanostructured materials, including nanotubes and two- dimensional nanomaterials, is being widely investigated as a model system for understanding nano–bio interfaces at the molecular level.^1–17^

The unique electronic and optical properties of nanotubes, such as their high surface area, exceptional mechanical strength, chemical stability under normal conditions, and biocompatibility, make them excellent platforms for investigating and leveraging their interactions with biomolecules, including nucleobases, proteins, lipids, carbohydrates, and organic drug molecules. The interactions between biomolecules and nanotubes have attracted considerable interest because they can substantially modify the structural, electronic, and optical properties of both the host nanomaterial and the guest molecule, leading to possible biotechnological applications.^18–21^ Recent studies have demonstrated that nanotube–nucleobase interactions can influence the electronic response of the nanotube and have therefore been explored in various applications.^19,22–24^ However, understanding the fundamental interaction mechanisms between nucleobases and nanotube surfaces remains an essential prerequisite for evaluating such possibilities.

Carbon nanotubes (CNTs) have long been explored extensively in this context. A large body of theoretical and experimental work has examined the interaction (adsorption and encapsulation) of DNA-related heterocyclic compounds and related biomolecules with CNTs, revealing that the interactions are primarily governed by dispersion forces, π–π stacking, and weak charge transfer effects.^1–3,7^ More recently, attention has also shifted toward BNNTs–which offer distinct electronic properties and chemical stability for similar host—guest interactions. Although CNTs and BNNTs share several common properties, BNNTs exhibit distinct features that provide unique advantages for various applications. Unlike CNTs, which can be metallic or semiconducting depending on their chirality and structure, BNNTs are basically insulators, independent of chirality and morphology, with minimal electron conduction under normal conditions due to their wide band gap.^25^ This makes BNNTs particularly suitable for applications requiring high thermal stability and low electronic interference, such as dielectric materials in electronics. The boron and nitrogen atoms have differing electronegativities, leading to partial ionic bonding and localized charge imbalances. This inherent polarity results in a chemically reactive surface ideal for surface functionalization and chemical modifications, such as attaching organic molecules, metal ions, or other functional groups. The polar nature also enhances the interaction with polar or ionic materials in case of encapsulation, thereby improving the compatibility and stability of the encapsulated entities. Moreover, as BNNTs exhibit superior chemical inertness and structural stability compared to CNTs, they are generally considered as nontoxic that enhances their potential for biomedical applications.^26–29^ Furthermore, BNNTs exhibit excellent piezoelectric properties, which CNTs lack.^22,30,31^ The combination of piezoelectric properties and polarized covalent bonds in BNNTs makes them highly suitable for novel energy harvesting applications.^31^

Consequently, several investigations have been performed on the interaction of biomolecules with BNNTs that include functionalization^6,7,27,32^ adsorption^5,33–36^ insertion^27^ and encapsulation.^37–44^ Theoretical investigations usually employ various computational methods, including density functional theory (DFT), molecular dynamics simulations, and quantum theory of atoms in molecules (QTAIM) analysis, to elucidate the encapsulation mechanisms and drug-nanotube interactions, providing valuable insights for the development of BNNT-based drug delivery systems. Comparative studies between CNTs and BNNTs reveal that BNNTs exhibit higher van der Waals interaction energies and more stable encapsulation of drugs like fluorouracil and azacitidine.^37,38^ Anticancer drugs like Floxuridine and Diethylstilbestrol have been shown to be effectively encapsulated by BNNTs through favorable adsorption energies and physisorption processes.^39,40^ The encapsulation of polyoxometalates in BNNTs has also been demonstrated, driven by Lewis acid–base interactions and photoactivated electron transfer.^41^ The performance of BNNT and hydroxylated BNNT as nanocarriers for platinum drugs was reported, with hydroxyl groups shown to facilitate drug insertion through hydrogen bonding and van der Waals interactions.^42^ Molecular dynamics simulations have further explored the wrapping of dinucleotides around CNTs,^45^ wrapping of polynucleotides,^46^ encapsulation and diffusion behavior of therapeutic molecules in BNNTs, providing insight into host–guest interaction mechanisms and transport properties.^42,43^

Although these studies have significantly advanced the understanding of BNNT–biomolecule interactions, considering the specific case of nucleobases, we observe that significant effort have been dedicated on adsorption of nucleobases on external surfaces of BNNTs. For example, Mukhopadhyay et al.^6^ examined the adsorption of the five canonical nucleobases on the outer wall of a high curvature semiconducting single-walled BNNT by first- principles DFT calculations, employing the plane wave pseudopotential approach within the local density approximation (LDA), and found that the interactions are predominantly noncovalent, with guanine exhibiting the strongest affinity among the nucleobases. More recently, Wang and Guo^8^ systematically investigated the exoadsorption of nucleobases on pristine and boron-rich BNNTs using localized-orbital quantum-chemical methods at the B97XD/6-311+G­(d,p)//B97XD/6-31G­(d,p) level. They demonstrated that boron-rich nanotubes exhibit substantially enhanced binding strengths due to the formation of B–N or B–O interactions accompanied by charge transfer and hydrogen-bond stabilization. In contrast, such systematic investigations on the nucleobase encapsulation in BNNTs remain relatively limited. On the other hand, we found that Monavari and Memarian,^1^ very recently, investigated the encapsulation of DNA nucleobases inside chiral CNTs, applying DFT based on tight binding method (DFTB) by considering semiempirical basis sets, demonstrating that confinement within nanotube cavities is energetically feasible and can induce measurable modifications in the structural and electronic properties of both the guest molecule and the host nanotube. These studies established the importance of nanotube diameter, chirality, and molecular orientation in determining host–guest interactions under confinement. This motivated us to examine the endohedral interaction of the nucleobases with BNNTs of different diameters and chiralities, aiming to elucidate the role of confinement on their structural, energetic, and electronic properties of these endocomplexes. However, it is important to note that isolated nucleobases are not biologically active entities outside the context of nucleosides or nucleotides. Nevertheless, they represent the fundamental aromatic and hydrogen-bonding units that determine the electronic and structural behavior of nucleic acids. Studying the interaction of individual nucleobases with nanostructured materials therefore provides a reductionist framework for isolating intrinsic interaction mechanisms before introducing the additional complexity associated with full nucleotides, polymers, or solvent environments.

In this study, we investigate the electronic properties of the nucleobases encapsulated within BNNTs. Although free nucleobases are not biologically active on their own, they are the fundamental building blocks of nucleosides and nucleotides, and studying their interaction with BNNTs provides insight relevant to more complex biomolecular interactions. Four BNNTs with different chirality and diameters were chosen: BNNT­(6,6), BNNT­(7,7), BNNT­(11,0) and BNNT­(12,0). The selected BNNT chiralities represent both armchair and zigzag configurations with varying diameters. Additionally, BNNTs with comparable diameters have been experimentally synthesized which motivated us to consider the above systems. The encapsulation of the five nucleobases in four BNNTs resulted in 20 distinct encapsulated systems. Section 2 of the manuscript exhibits the computational details and relevant technical information on the doped heterostructures. Section 3 presents the results and discussion. A brief summary and conclusion have been provided in Section 4.

## 2 Calculation Details

The structural and electronic properties of the encapsulated BNNTs were investigated to assess the effect of adsorption of the nucleobases. The calculations were performed using Density Functional Theory (DFT), as implemented in the ab initio Quantum Espresso (QE) code,^47^ employing ultrasoft pseudopotentials (USPP)^48^ and a plane-wave basis set, extensively used for extended nanostructures.^49–53^ The exchange-correlation effects were treated within the generalized gradient approximation (GGA) using the PBE functional.^54^ To account for van der Waals interactions, Grimme’s semiempirical DFT-D2 method was applied.^55,56^ The BNNT models used in our study are treated under periodic boundary conditions^57–60^ as implemented in Quantum ESPRESSO, and therefore do not contain finite edges or terminal atoms. As a result, artificial edge states arising from dangling bonds are not present in our calculations. The encapsulated nucleobases interact with an effectively infinite nanotube along the axial direction, avoiding the need for chemical termination (H or OH). The pristine nanotube supercells considered in the encapsulation process contain 96, 112, 132, and 144 atoms in BNNT­(6,6), BNNT­(7,7), BNNT­(11,0) and BNNT­(12,0), respectively. The respective lengths of the supercell for the above chiralities are 9.85 Å, 8.61 Å, 11.38 Å and 11.38 Å. The kinetic energy cutoff was set to 0.3 keV, while the charge density cutoff was fixed at 3 keV. The Brillouin zone was sampled using a 1 × 1 × 36 Monkhorst–Pack k-point mesh^61^ which weel describes the binding and the electronic properties of the systems. For structural relaxations, the convergence criterion for forces and total energy were set to 10^–3^ Ry/Bohr, and 10^–4^ Ry, respectively. The visualization of the structural models of all the encapsulated systems were performed using the Xcrysden^62^ and Vesta^63^ packages. The formation energy per atom *E*_*form*_ which determines whether the above encapsulation is favorable under equilibrium conditions, is given by

1 $$E_{form}=\left(E_{BNNT+NB}-E_{BNNT}-E_{NB}\right)/n$$

where *E*_BNNT+NB_ is the energy of the encapsulated systems, *E*_*BNNT*_ and *E*_*NB*_ are the energies of the pristine nanotubes and pristine nucleobases, respectively, *n* is the number of atoms of the unit cell. All the structures were fully optimized to study the effect of the encapsulation on the structural characteristics and the electronic properties of the encapsulated BNNTs with nucleobases. During the optimization process, the nucleobases were initially aligned parallel to the BNNT growth direction and positioned at the center of the supercell.

## 3 Results and Discussion

The impact of the adsorption of the nucleobases on the electronic and structural properties of the BNNTs with different chiralities has been addressed using the first-principles calculations. Figure 1 exhibits the perspective of the optimized structures of encapsulated systems with the nucleobases in the BNNTs along the nanotube growth direction. In Figure 1, B, N, H, O, and C atoms are indicated in green, blue, white, red and black, respectively. The distances along the *x* and *y* axes have been adequately chosen so as to avoid interactions between the layers. BNNT­(6,6) and BNNT­(7,7) have arm-chair configurations whereas BNNT­(11,0) and BNNT­(12,0) have zigzag configurations. The diameters of the pristine nanotubes are 8.34 Å, 9.75 Å, 8.79 Å and 9.59 Å for the above four chiralities, respectively. The encapsulation of the nucleobases leads to a strain in the nanotube structures leading to a modification of the B–N bond lengths, thereby altering the perfect circular nature of the nanotubes. Unlike the circular nature of the pristine nanotubes, encapsulated ones exhibit a elliptic nature. Compared to the pristine nanotubes the major modification is observed in the encapsulated BNNT­(6,6) which has the smallest diameter among the four in this study, followed by BNNT­(11,0). Among the nucleobases, guanine encapsulation leads to the highest deformation of the nanocarrier whereas the least deformation is observed for uracil which could be explained by the number of its constituent atoms. It is interesting to note that (12,0) preserves the circular nature and the diameter of the nanotubes, whereas BNNT­(7,7) shows deformation for adenine and guanine encapsulation. BNNT­(6,6) exhibits the greatest deformation, reaching up to 13% for adenine and guanine and approximately 7% for cytosine and uracil. In case of BNNT­(7,7) the largest variation occurs with adenine and guanine, around 5%, while the other nucleobases show no significant changes. In the case of BNNT­(11,0) the variations are smaller compared to those observed in BNNT­(6,6). It is of the order of nearly 8% in adenine and guanine encapsulation and is around 4% in cytosine and uracil enacpsulation.

**Figure 1:**
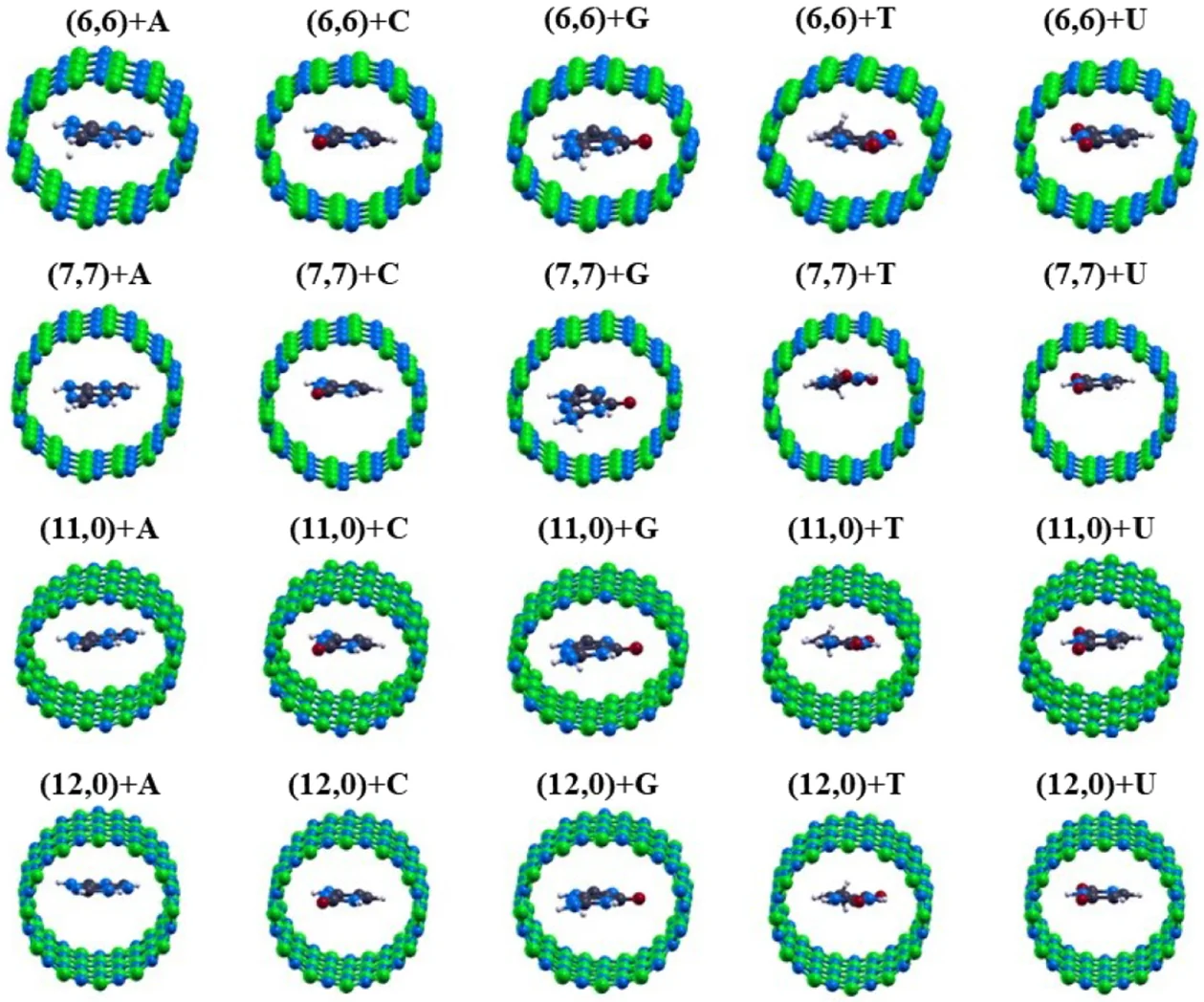
Optimized structures of the encapsulated systems involving the nucleobases in BNNT­(6,6), BNNT­(7,7), BNNT­(11,0) and BNNT­(12,0). Each structure is identified by its chirality and the type of encapsulated nucleobase. B, N, H, O, and C atoms are indicated in green, blue, white, red and black, respectively.

In order to understand the stability and the possibility of the formation of the above encapsulated systems, the formation energies (*E*_*form*_) for the 20 systems are exhibited in Table 1. The negative values of the formation energies demonstrate the possibility of encapsulation of the nucleobases in all the nanotubes chosen, irrespective of their chirality or diameter. So strong physical adsorption has been observed between the nucleobases and the nanotube as was also observed in CNTs^1,3,10,64–66^ Moreover, we do not observe any structural deformation of the nucleobases. Hence the preservation of the nucleobases during the encapsulation process is evident. Among the four types of nanotubes, while BNNT­(6,6) shows the lowest value of the formation energy, BNNT­(12,0) exhibits the highest value. The arm-chair nanotubes has a lower value of the formation energy when compared to the zigzag formats. It is interesting to note that *E*_*form*_ has nearly no dependence on the type of nucleobase.

**Table 1:** Structural Data and the Formation Energies of the Encapsulated BNNT Systems

	Adenine			Cytosine			Guanine			Thymine			Uracil		
Chirality	*E_form_* (eV)	*Egap* (eV)	Δ*E*_gap_ (eV)	*E_form_* (eV)	*E*_gap_ (eV)	Δ*E*_gap_ (eV)	*E_form_* (eV)	*E*_gap_ (eV)	Δ*E*_gap_ (eV)	*E_form_* (eV)	*Egap* (eV)	Δ*E*_gap_ (eV)	*E_form_* (eV)	*Egap* (eV)	Δ*E*_gap_ (eV)
(6,6)	–0.076	3.54	0.50	–0.080	3.30	0.74	–0.075	3.60	0.45	–0.080	3.29	0.76	–0.082	4.14	0.90
(7,7)	–0.073	3.55	0.57	–0.073	3.28	0.84	–0.074	3.75	0.37	–0.074	3.27	0.85	–0.074	3.15	0.97
(11,0)	–0.009	3.56	0.54	–0.009	3.25	0.85	–0.009	3.75	0.36	–0.010	3.26	0.85	–0.009	3.12	0.98
(12,0)	–0.008	3.64	0.51	–0.007	3.31	0.83	0.008	3.76	0.38	–0.008	3.25	0.90	–0.007	3.09	1.05

The band gap (*E*_*gap*_) of the pristine nanotubes as well as that of the encapsulated ones were calculated so as to understand the electronic nature of the encapsulated systems. BNNT is a wide gap insulator and on encapsulation the systems demonstrate a decrease in the band gap values for all the nucleobases which was calculated using Δ*E*_gap_. Hence the encapsulation should lead to modified optical properties. The values of Δ*E*_gap_ do not depend on the chirality or the diameter of the nanotubes but is dependent on the type of nucleobase as was also observed in the work of Monavari et al.^1^ The highest value of Δ*E*_gap_ is observed for the RNA nucleobase. Among the nucleobase the highest value is for cytosine and thymine. The encapsulation of the nucleobases in CNTs led to a modification of gap between 0 and 0.24 eV,^1^ whereas that in BNNTs leads to a modification as high as 0.9 eV. Moreover the encapsulation of uracil leads to an even higher modification of more than 1.0 eV. The encapsulation of the A and T nucleobases inside CNT­(7,6) also did not cause significant change in the band gap values.^10^

Upon analyzing the Lowdin charges of the N and B atoms we observe that there has been a increase of the charges of the B atoms whereas a decrease in that of the nitrogen atoms. The changes vary depending on the position of the atoms in the nanotubes. The maximum change in B­(N) atoms is of the order of +0.025e (−0.006e). The variation in the Lowdin charges verifies the encapsulation process of the nucleobases.

Figure 2 displays the HOMO and LUMO isosurfaces for the sequencing purpose of the five nucleobases–adenine (A), cytosine (C), guanine (G), thymine (T), and uracil (U) encapsulated inside boron nitride nanotubes (BNNTs) of different chiralities: (6,6), (7,7), (11,0), and (12,0). The spatial distribution of the molecular orbitals indicates the electronic interaction between the nucleobases and the nanotube walls. The positive and negative distributions are denoted in blue and yellow. The presence of the electronegative ions tends to modify the HOMO and LUMO surfaces of the different nucleobases.

**Figure 2:**
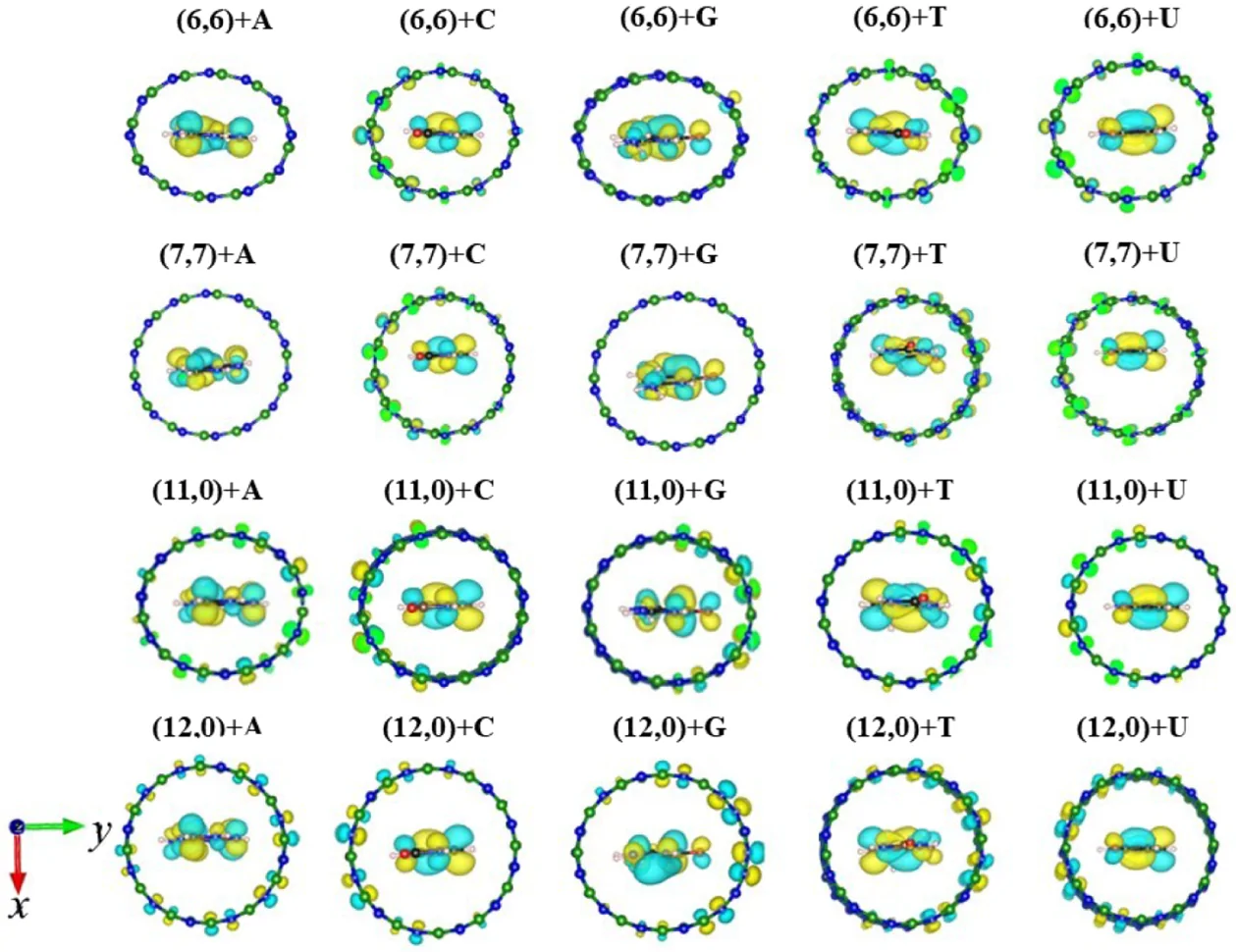
HOMO–LUMO of the encapsulated BNNTs with adenine, cytosine, guanine, thymine and uracil.

Figure 3 presents the energy band structures along the Γ–Z path and the projected density of states (PDOS) for the pristine nanotubes with chiralities (6,6), (7,7), (11,0), and (12,0). The gap of the nanocarriers is 4.1 eV approximately. The increase in the number of atoms in the supercell gives rise to the increase in the PDOS. While the contribution from the nitrogen atoms plays a dominant role in the valence band, whereas that from the boron atoms prevails in the conduction band.

**Figure 3:**
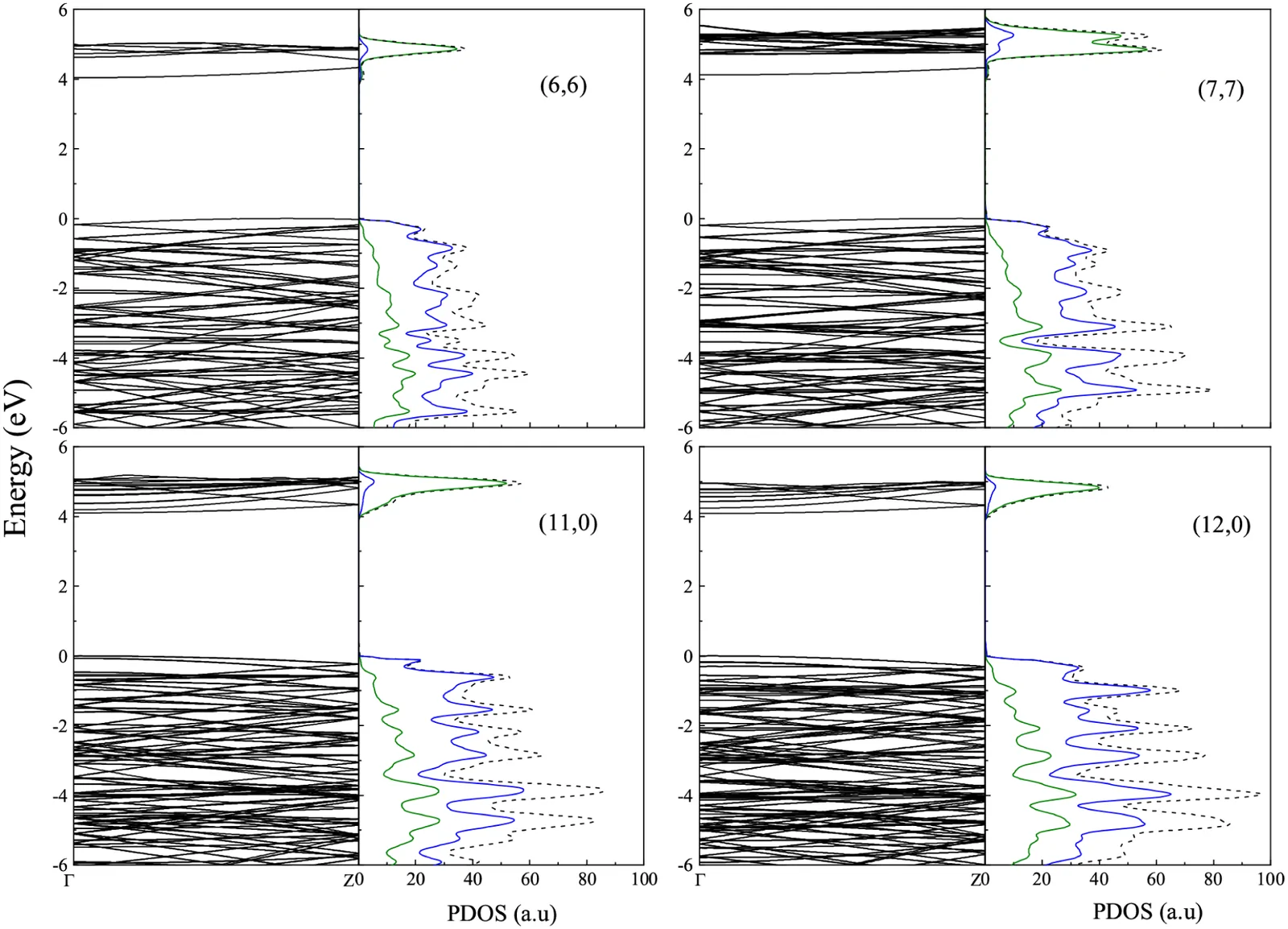
Energy-bands and the projected density of states of the pristine (6,6), (7,7), (11,0) and (12,0) BNNTs. The Fermi energy of the systems has been put as zero in the figures.

Figure 4 displays the energy-band structures of all the encapsulated BNNTs along the path Γ-Z. All the figures are labeled by the chirality of the nanotube and also the type of nucleobase utilized. The energy-band structures of encapsulated systems exhibit significant modification due to interactions and strain effects introduced by the encapsulating material. The band gap of the systems is evident from the above figures and the modifications in their values are helpful for the band gap engineering. Moreover, we observe that the presence of nucleobases leads to an increase in the number of bands in the energy range considered here. The decrease in the band gap could be explained by the energy levels created by the nucleobases close to the Fermi energy. Moreover the increase in the number of energy levels should also increase the density of states of the encapsulated systems.

**Figure 4:**
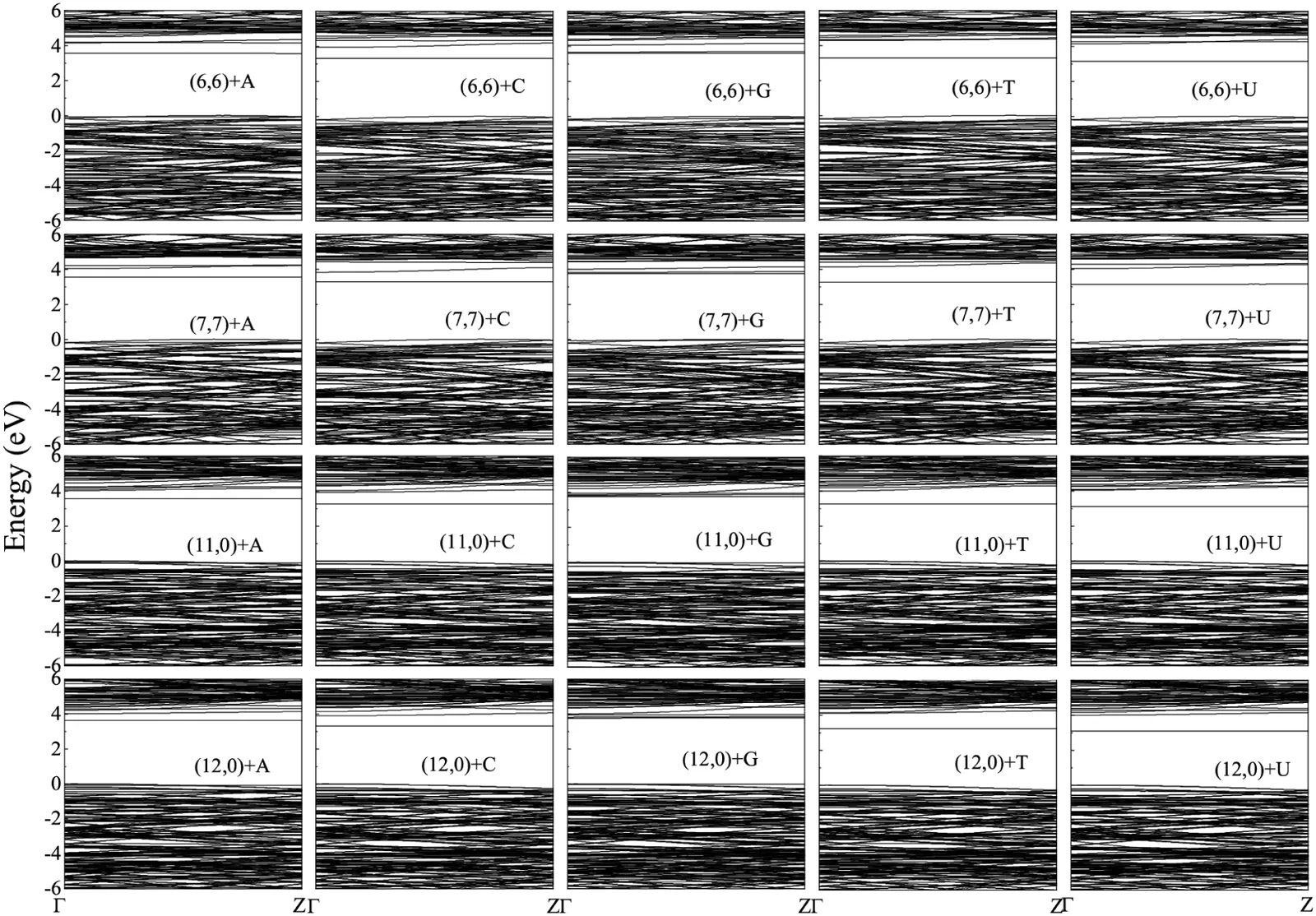
Energy-bands of the encapsulated BNNTs with adenine, cytosine, guanine, thymine and uracil. The Fermi energy of the systems has been put as zero in the figures.

The projected density of states (PDOS) of the encapsulated systems are exhibited in Figure 5. The individual atomic contributions to the PDOS are shown, with B (green), N (blue), C (black), H (orange), and O (red). The increase in the supercell size leads to an increase in the total PDOS of the systems. The contribution are given in the following order (12,0) > (11,0) > (7,7) > (6,6). The contribution from the nitrogen atoms are considerably higher when compared to that of the boron atoms in the valence band of all the encapsulated BNNTs. However, in the conduction band of the systems the contribution from the boron atoms prevails with very little contribution from that of the nitrogen atoms. The decrease of the band gap in all the systems are caused mainly due to the states that are created in the conduction band by the nitrogen and the carbon atoms of the nucleobases irrespective of the chirality of the tubes. The contribution of the oxygen atoms of the nucleobases are more dominant in the valence band of the systems.

**Figure 5:**
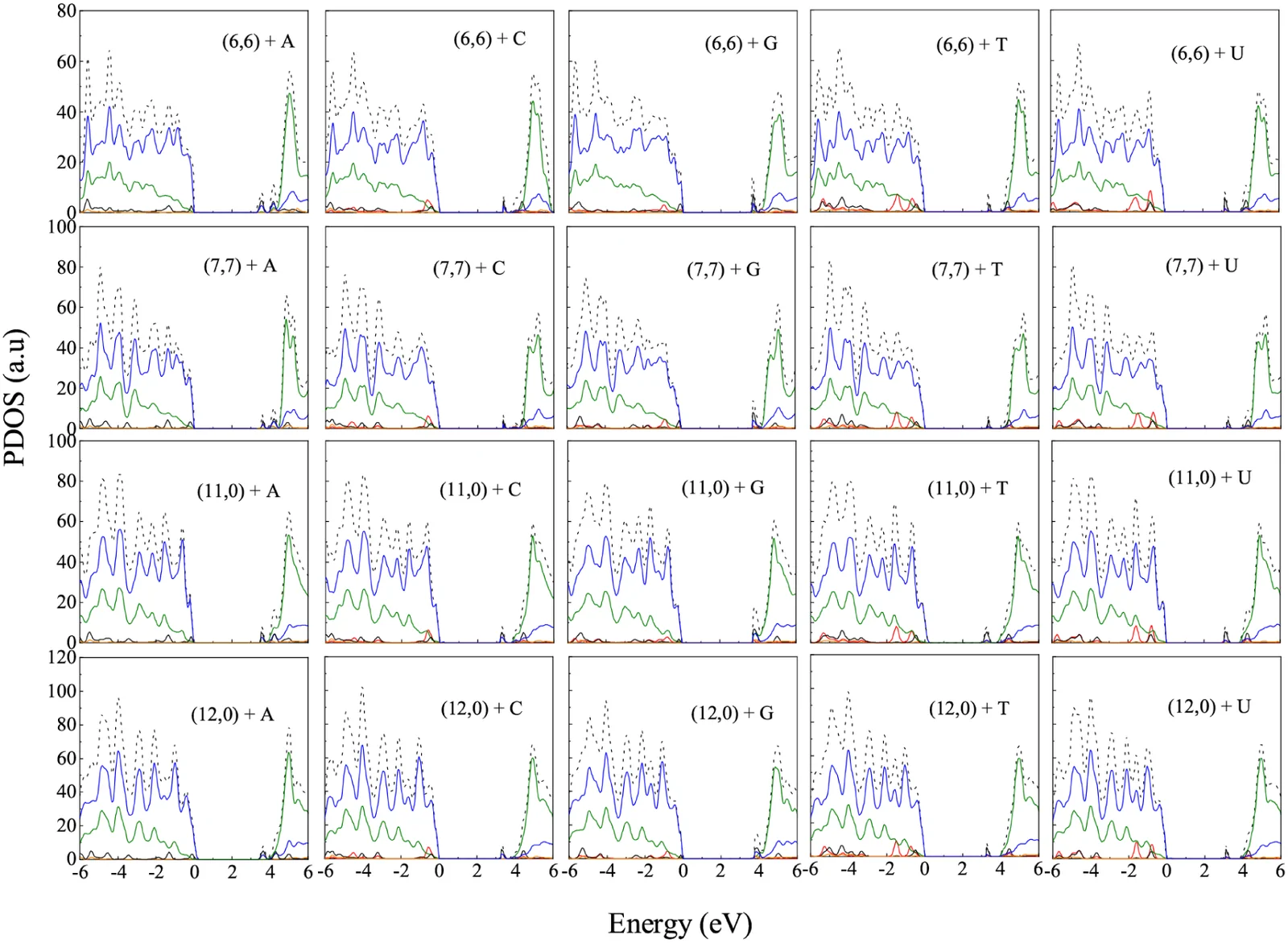
Total projected density of states and the contribution of the atoms for the encapsulated systems.

## 4 Conclusion

The encapsulation of nucleic-acid-derived aromatic heterocycles (nucleobases) inside the boron nitride nanotubes (BNNT) was investigated using density functional theory calculations. Structural and electronic properties of 20 different optimized system resulting out of the encapsulation of five heterocycles (adenine, cytosine, guanine, thymine, and uracil) in the zigzag (11,0) and (12,0) and the armchair (6,6) and (7,7) BNNTs were calculated. The formation energies indicate that encapsulation is energetically favorable for all heterocycle–BNNT systems considered, demonstrating the stability of the resulting host–guest complexes. Structural analysis revealed that confinement effects are strongly dependent on nanotube diameter, with the smaller BNNT­(6,6) exhibiting the largest deformation upon encapsulation. These geometric modifications are accompanied by measurable changes in the electronic structure of the nanotubes. Analysis of the energy-band diagrams and density of states showed that the encapsulated heterocycles introduce electronic states in the vicinity of the Fermi level, resulting in systematic band gap reduction relative to the pristine nanotubes. Among the systems investigated, adenine exhibits the most favorable formation energy, while uracil produces the largest band gap modification irrespective of nanotube chirality. The results demonstrate that molecular confinement inside BNNT cavities can significantly influence both the structural stability and electronic properties of the host nanotubes. More broadly, the present study provides fundamental insight into confinement-driven host–guest interactions between aromatic heterocycles and boron nitride nanotubes, contributing to the understanding of nanoscale encapsulation phenomena in low-dimensional materials.

## Data Availability

Data generated and analyzed in the present study are available upon request.
